# Supplementary material for: Using LLMs and Explainable ML to Analyze Biomarkers at Single-Cell Level for Improved Understanding of Diseases
Source: Biomolecules. 2023 Oct 12;13(10):1516. doi: 10.3390/biom13101516 (PMC10605495; doi:10.3390/biom13101516)
Supplement: Supplementary file 1 [file biomolecules-13-01516-s001.zip › biomolecules-2601544-supplementary.pdf]

Using LLM Models and Explainable ML to Analyse Biomarkers at Single Cell Level for Improved Understanding of Diseases

Jonas Elsborg[1, 2, +] and Marco Salvatore[2,+, *]

[1]Department of Energy Conversion and Storage, Technical University of Denmark, 2800 Kongens Lyngby, Denmark
[2]Abzu ApS, Orient Plads, Copenhagen, 2150, Denmark
[*]marco.salvatore@abzu.ai
[+]these authors contributed equally to this work

September 26, 2023

Keywords— biomarker, LLM, interpretability, scRNA-seq, machine learning, symbolic regression

A Supplementary Information

Train ID	Test ID	Cell Type	Dominant Features	PR AUC	Score	Count	UC Fraction
D1	D5	Cycling B cell	CHI3L2,ESCO2	1.0/0.98	0.97	13/77	38%/3%
D1	D2	Pericyte	RPL39, COL6A3	0.97/0.96	0.96	1039/260	13%/8%
D1	D2	arterial capillary	RPL39,PLAT	0.95/0.94	0.94	1814/559	28%/22%
D1	D2	Mature venous EC	RPL39,PLAT	0.96/0.94	0.93	2457/713	15%/13%
D1	D3	Goblet cell	LYZ, LGALS4	0.94/0.92	0.91	1714/325	26%/38%
D1	D2	Stromal 4(MMP1+)	LGALS1,IGFBP 3	1.0/0.94	0.91	1111/569	1%/7%
D1	D3	Stem cells	CD74,ERRFI1	0.96/0.92	0.90	123/102	21%/19%
D1	D2	Mature arterial EC	RPL39,PLAT	0.96/0.92	0.90	702/118	15%/18%
D1	D3	IgA plasma cell	LCN2,FABP1	0.92/1.0	0.88	1182/46	25%/4%
D1	D3	TA	B3GNT7,FABP1	0.95/0.89	0.86	14930/2821	35%/36%
D1	D3	cDC2	LCN2,HMGB2	0.87/0.91	0.85	81/11	21%/18%
D1	D5	Contractile pericyte (PLN+)	CAV1,APOE	0.89/0.86	0.84	277/11	17%/27%
D1	D3	BEST 2+ Goblet cell	LYZ, FABP1	0.96/0.88	0.84	11444/1436	24%/42%
D1	D3	EC cells (TAC1+)	FABP1,HEXIM1	0.97/0.86	0.80	125/66	19%/26%
D1	D3	Colonocyte	B3GNT7,LCN2	0.87/0.82	0.80	51235/4815	43%/42%
D1	D3	BEST4+ epithelial	DST,AGR2	0.89/0.82	0.78	2801/299	34%/35%
D1	D2	myofibroblast	TM4SF1,RPL39	0.97/0.82	0.74	1189/105	16%/45%
D1	D2	Fetal arterial EC	ARRB2,PASK	0.98/0.82	0.74	63/15	5%/27%
D1	D2	Adult Glia	RPL39,SCN7A	0.79/0.75	0.73	1478/617	45%/42%
D1	D2	Contractile pericyte (PLN+)	CAV1,APOE	0.89/0.75	0.68	277/243	17%/27%

Table 1: Summary of the performance of all obtained models for Dataset 1 on different cell types. This table provides a comprehensive overview of our model's performance across diverse cell types, employing various features and evaluating through PR AUC. The "Dominant Features" column highlights the gene that consistently emerges as the most frequent among the 10 models generated by the QLattice. The "Score" column in the table represents the transferability-corrected performance score explained in details in the method section. Furthermore, the "UC Fraction" column delineates the proportion of cell types corresponding to disease samples within both the training and test datasets.

Train ID	Test ID	Cell Type	Dominant Features	PR AUC	Score	Count	UC Fraction
D2	D1	mLTo	MT2A,C7	1.0/1.0	1.00	37/185	49%/2%
D2	D1	myofibroblast (RSPO2+)	CXCL1, IF16	1.0/0.98	0.97	10/294	40%/3%
D2	D1	Stromal 4(MMP1+)	RPS4Y1,IFI27	0.97/1.0	0.96	569/1111	7%/1%
D2	D1	Fetal arterial EC	GJA4,IFI27	1.0/0.97	0.96	15/63	27%/5%
D2	D1	Fetal venous EC	TM4SF1,COL6A2	1.0/0.96	0.94	11/13	36%/23%
D2	D1	Stromal 3(C7+)	CXCL1,RPS $4Y1$	0.99/0.96	0.94	18/347	33%/4%
D2	D1	myofibroblast	RPS 4 Y1,APOC1	0.94/0.92	0.91	105/1189	45%/16%
D2	D1	T reticular	IFI27, CLU	0.92/0.9	0.89	75/2101	49%/20%
D2	D1	Stromal 1 (ADAMDEC1+)	CFD,RPS 4Y1	0.96/0.9	0.87	219/5626	23%/28%
D2	D1	Pericyte	RPS 4Y1, IFI27	0.96/0.88	0.84	260/1039	8%/13%
D2	D1	Mature arterial EC	RPS4Y1,HLA-DRB1	0.95/0.87	0.83	118/702	18%/15%
D2	D1	Mature venous EC	RPS $4Y1, C7$	0.95/0.85	0.80	713/2457	13%/15%
D2	D1	Contractile pericyte (PLN+)	IFI27, FABP4	0.97/0.86	0.80	243/277	27%/17%
D2	D5	Contractile pericyte (PLN+)	IFI27, FABP4	0.97/0.83	0.76	243/11	27%/27%
D2	D1	Stromal 1(CCL11+)	RPS 4 Y 1,CFD	0.93/0.82	0.76	2479/5193	26%/36%
D2	D1	Transitional Stromal 3(C3+)	PDLIM3,TNFRSF12A	0.74/0.85	0.68	30/56	7%/23%
D2	D1	arterial capillary	RPS4Y1, CABP1	0.95/0.75	0.65	559/1814	22%/28%
D2	D1	Stromal 2(NPY+)	PLAT,RPS 4Y1	0.96/0.74	0.63	2584/6813	43%/35%
D2	D1	Adult Glia	CD74,TNFSF13B	0.99/0.63	0.45	617/1478	42%/45%
D2	D5	IgA plasma cell	CXCL1,HLA-DRB1	0.51/0.43	0.39	460/7597	5%/42%

Table 2: Summary of the performance of all obtained models for Dataset 2 on different cell types. This table provides a comprehensive overview of our model's performance across diverse cell types, employing various features and evaluating through PR AUC. The "Dominant Features" column highlights the gene that consistently emerges as the most frequent among the 10 models generated by the QLattice. The "Score" column in the table represents the transferability-corrected performance score explained in details in the method section. Furthermore, the "UC Fraction" column delineates the proportion of cell types corresponding to disease samples within both the training and test datasets.

Train ID	Test ID	Cell Type	Dominant Features	PR AUC	Score	Count	UC Fraction
D3	D1	BEST2+ Goblet cell	LCN2,FABP1	0.92/0.94	0.91	1436/11444	42%/24%
D3	D1	TA	FABP1,LCN2	0.93/0.91	0.90	2821/14930	36%/35%
D3	D1	Stem cells	LCN2,DDIT4	0.92/0.88	0.86	102/123	19%/21%
D3	D5	Treg	CCL5,CD69	0.96/0.87	0.82	121/4304	8%/15%
D3	D1	EC cells (TAC 1+)	RPS $4Y1$, $MT1G$	0.91/0.85	0.82	66/125	26%/19%
D3	D1	Goblet cell	LCN2, S100P	0.94/0.84	0.79	325/1714	38%/26%
D3	D5	Naive B	HSPA2,GPM6B	1.0/0.84	0.76	41/550	2%/17%
D3	D5	Tfh	FAM43A,FAM167B	1.0/0.83	0.74	25/53	4%/19%
D3	D5	gdT	LGALS1,HLA-DPA1	0.89/0.78	0.72	86/293	35%/28%
D3	D1	Colonocyte	CKB,LCN2	0.9/0.78	0.72	4815/51235	42%/43%
D3	D1	CDC2	FABP1,NLRP3	1.0/0.81	0.72	11/81	18%/21%
D3	D5	TRGV4 gdT	ZFP36L2, BIRC3	1.0/0.79	0.68	27/57	48%/30%
D3	D5	Memory B	ABHD5,IGFBP2	0.96/0.75	0.64	183/3139	5%/25%
D3	D5	NK cell	RPS 4 Y 1,KLRB1	0.95/0.74	0.64	78/831	46%/31%
D3	D1	IgA plasma cell	KLF2,MT1M	0.99/0.76	0.64	46/1182	4%/25%
D3	D5	SELL + CD4 T	NR4A2, NABP1	1.0/0.75	0.62	12/2739	17%/26%
D3	D1	BEST4+ epithelial	ISG20,S100P	0.93/0.69	0.57	299/2801	35%/34%
D3	D5	CDC2	FABP1,NLRP3	1.0/0.62	0.43	11/192	18%/43%
D3	D5	Activated CD8 T	CCL5,NR4A2	0.9/0.58	0.42	460/3212	43%/41%
D3	D5	Mast cell	RPS4Y1,SAMSN1	1.0/0.51	0.26	26/16	38%/31%

Table 3: Summary of the performance of all obtained models for Dataset 3 on different cell types. This table provides a comprehensive overview of our model's performance across diverse cell types, employing various features and evaluating through PR AUC. The "Dominant Features" column highlights the gene that consistently emerges as the most frequent among the 10 models generated by the QLattice. The "Score" column in the table represents the transferability-corrected performance score explained in details in the method section. Furthermore, the "UC Fraction" column delineates the proportion of cell types corresponding to disease samples within both the training and test datasets.

Train ID	Test ID	Cell Type	Dominant Features	PR AUC	Score	Count	UC Fraction
D5	D3	Treg	RPL39,CD7	0.95/0.98	0.94	4304/121	15%/8%
D5	D3	Tfh	DDIT4,PPP1R3E	0.89/0.98	0.85	53/25	19%/4%
D5	D3	Memory B	RPS4Y1,ADAM28	0.87/0.97	0.82	3139/183	25%/5%
D5	D3	Naive B	RPS4Y1,GEM	0.88/0.99	0.82	550/41	17%/2%
D5	D3	gdT	RPL39,CD74	0.93/0.86	0.82	293/86	28%/35%
D5	D3	Activated CD8 T	RPL39,KLF2	0.82/0.84	0.81	3212/460	41%/43%
D5	D1	cDC2	LYZ,RPL39	0.81/0.83	0.80	192/81	43%/21%
D5	D3	SELL+ CD4 T	CCL5,CD3D	0.85/0.96	0.80	2739/12	26%/17%
D5	D1	IgA plasma cell	RPL39,CD79A	0.85/0.81	0.79	7597/1182	42%/25%
D5	D3	IgA plasma cell	RPL39,CD79A	0.85/1.0	0.78	7597/46	42%/4%
D5	D3	TRGV4 gdT	CD8A,NR4A2	0.98/0.83	0.76	57/27	30%/48%
D5	D1	Contractile pericyte (PLN+)	MFGE8,CFD	1.0/0.84	0.76	11/277	27%/17%
D5	D1	Activated T	GZMA,CD74	0.88/0.78	0.73	757/37	30%/49%
D5	D3	CDC2	LYZ,RPL39	0.81/0.98	0.72	192/11	43%/18%
D5	D3	Activated CD4 T	RPL39,KLF2	0.76/0.86	0.71	6154/39	38%/31%
D5	D2	Contractile pericyte (PLN+)	MFGE8,CFD	1.0/0.78	0.67	11/243	27%/27%
D5	D3	NK cell	RPL39,GZMB	0.9/0.64	0.51	831/78	31%/46%
D5	D3	Mast cell	STMN1,ZFP36L2	0.94/0.63	0.47	16/26	31%/38%
D5	D1	Cycling B cell	SYTL2,MZB1	0.99/0.42	0.14	77/13	3%/38%
D5	D2	IgA plasma cell	RPL39,CD79A	0.85/0.18	-0.16	7597/460	42%/5%

Table 4: Summary of the performance of all obtained models for Dataset 4 on different cell types. This table provides a comprehensive overview of our model's performance across diverse cell types, employing various features and evaluating through PR AUC. The ”Dominant Features” column highlights the gene that consistently emerges as the most frequent among the 10 models generated by the QLattice. The ”Score” column in the table represents the transferability-corrected performance score explained in details in the method section. Furthermore, the ”UC Fraction” column delineates the proportion of cell types corresponding to disease samples within both the training and test datasets.
